# Special Issue “The Past and Present Threat of Rickettsial Diseases”

**DOI:** 10.3390/tropicalmed5040187

**Published:** 2020-12-16

**Authors:** Daniel H. Paris

**Affiliations:** 1 Swiss Tropical and Public Health Institute, 4051 Basel, Switzerland; daniel.paris@swisstph.ch; 2 Department of Clinical Research, University of Basel, 4051 Basel, Switzerland

Historically, the rickettsioses have a track record of making substantial impact on mankind in military activities and international public health over the past centuries [1]. Napoleon’s Grande Armée suffered from great losses due to epidemic typhus (and trench fever), while scrub typhus was the major infectious disease associated with losses in both Allied and Japanese troops in the Asia-Pacific region during World War II, and similarly during the Vietnam conflict. The discovery of chloramphenicol in the 1940s led to a sense of security in the treatment of these diseases and a subsequent reduction in related research activities.

Over the past decade, numerous “causes of febrile illness” studies have highlighted the growing global burden of febrile diseases, and revealed the tropical forms of rickettsial illnesses as prominent causes of treatable febrile illnesses in regions of SE-Asia—and suggesting a distribution along the global tropical and subtropical belts.

Tropical rickettsial illnesses are severely neglected infectious diseases responsible for a large burden of disease in both rural and urban populations, especially in Asian low- and middle-income countries. Scrub typhus and murine typhus have been described in tropical and subtropical regions for decades, but they remain under appreciated and scientifically neglected, despite the growing evidence for an ever-widening impact. The recent discovery of their expanding occurrence could be associated with “urban growth”, i.e., expansion of cities encroaching into rural areas; a wider use of adequate diagnostics due to “fever studies”; the discontinuation of chloramphenicol in the 1990s, which could have treated more hidden or “mis-diagnosed” rickettsioses than previously thought; an undocumented expansion or redistribution of infected vectors; and/or associations with climate change, etc. Clearly, many questions and speculations arise when diseases emerge in unexpected geographic regions outside their previously described areas of endemicity, and after decades of “silence” in regions where they had once been endemic [2].

Scrub typhus is likely the most relevant rickettsiosis in terms of worldwide attributed morbidity and mortality. In countries where established surveillance systems have incorporated scrub typhus (currently South Korea, Japan, China, and Thailand), a dramatic increase in minimum incidence rates over the past decade has been recorded [3]. Emerging reports from South America and Africa suggest a wider distribution beyond Asia, and improved diagnostics are contributing to a better understanding of the clinical epidemiology. Murine typhus is an equally important and even more neglected disease with an urban distribution pattern. Unfortunately, a lot of clinically relevant information on the natural history, immunology, diagnostics, and strain characterisation are lacking for both diseases.

Highlights in this Special Issue include updates to the global distribution of these illnesses, including the expansion of highly populous areas into rural areas, which has resulted in increased human exposure to vectors and zoonotic diseases [4]. Further, recent advances in diagnostics and the associated obstacles are discussed; despite the positive impact of molecular assays, major difficulties remain in validating new tests, which hamper the development of simple and inexpensive approaches for rural areas in low- and middle-income countries [5].

Our entomological knowledge gaps remain substantial—especially on the transmission aspects of mites and fleas—which represent a major obstacle for the design of novel control measures. New methodologies enabling paired morpho- and genotyping for mites have opened up important research avenues in vector biology and related ecological issues [6]. As the vectors also have a role as reservoir hosts, the issues of vector competence, identification of alternate reservoirs, understanding the change in sex ratios of infected mite populations, and designing environmental interventions (i.e., insecticides, etc.) represent important areas for future investigations.

Modern genome sequencing technologies and more in-depth immunological research are now needed more than ever to fill critical knowledge gaps; these would ideally target the characterisation of the (vast) strain diversity, understanding why natural immune protection is so short-lived, and advanced antigen discovery. All of these issues are pre-requisites for the development of a protective vaccine, and although the first promising results are encouraging, more research and development is required in these areas [7].

In this Special Issue of “Tropical Medicine and Infectious Disease”, we collated reviews, reports, and original research articles to identify open questions from the past as well as the present—as a way to look forward. This diverse collection of publications emphasises the need for enhanced diagnostic developments, in-depth epidemiological studies (beyond Southeast Asia), understanding relevant entomology and ecology interactions, dissecting the immune response (especially memory functions), identifying protective antigens and characterizing novel diagnostic targets.

I am most grateful for the passion and great perseverance shown by colleagues, rickettsiologists and scientists from all around the world for their inspiring work with these fascinating diseases. Your contributions made this Special Issue—comprising over 20 publications on tropical rickettsial diseases—truly special and I am confident that it will contribute to an improved awareness of these neglected but important diseases in physicians and researchers alike.
